# Single-cell imaging of phosphorus uptake shows that key harmful algae rely on different phosphorus sources for growth

**DOI:** 10.1038/s41598-018-35310-w

**Published:** 2018-11-21

**Authors:** Niels J. Schoffelen, Wiebke Mohr, Timothy G. Ferdelman, Sten Littmann, Julia Duerschlag, Mikhail V. Zubkov, Helle Ploug, Marcel M. M. Kuypers

**Affiliations:** 1 Department of Biogeochemistry, Max Planck Institute for Marine Microbiology, Celsiusstraße 1, 28359 Bremen, Germany; 2 Ocean Biogeochemistry and Ecosystems, National Oceanography Centre Southampton, European Way, Southampton, SO14 3ZH United Kingdom; 3 Department of Marine Sciences, University of Gothenburg, Carl Skottsbergs Gata 22B, 41319 Gothenburg, Sweden; 4 Present Address: Scottish Association for Marine Science, Oban, Argyll PA37 1QA Scotland, United Kingdom

## Abstract

Single-cell measurements of biochemical processes have advanced our understanding of cellular physiology in individual microbes and microbial populations. Due to methodological limitations, little is known about single-cell phosphorus (P) uptake and its importance for microbial growth within mixed field populations. Here, we developed a nanometer-scale secondary ion mass spectrometry (nanoSIMS)-based approach to quantify single-cell P uptake in combination with cellular CO_2_ and N_2_ fixation. Applying this approach during a harmful algal bloom (HAB), we found that the toxin-producer *Nodularia* almost exclusively used phosphate for growth at very low phosphate concentrations in the Baltic Sea. In contrast, the non-toxic *Aphanizomenon* acquired only 15% of its cellular P-demand from phosphate and ~85% from organic P. When phosphate concentrations were raised, *Nodularia* thrived indicating that this toxin-producer directly benefits from phosphate inputs. The phosphate availability in the Baltic Sea is projected to rise and therefore might foster more frequent and intense *Nodularia* blooms with a concomitant rise in the overall toxicity of HABs in the Baltic Sea. With a projected increase in HABs worldwide, the capability to use organic P may be a critical factor that not only determines the microbial community structure, but the overall harmfulness and associated costs of algal blooms.

## Introduction

Phosphorus (P) is an essential element for the growth of all living organisms, and the availability of P, in particular dissolved inorganic P (DIP, i.e. phosphate) limits productivity in a variety of ecosystems. The availability of DIP can play a vital role in the formation of harmful algal blooms in freshwater and brackish ecosystems^1,2^ and limit marine primary productivity^3^ as well as dinitrogen (N_2_) fixation^4^, the largest external input of nitrogen (N) to the ocean^5^. Microorganisms have developed a suite of features to cope with DIP scarcity, including adjustments of the cellular P requirements such as the replacement of phospholipids with sulfur-based lipids^6^, the intracellular storage of P for use at times of P starvation^7^, and the direct use of organic P sources^8,9^. In addition, organic P sources could be exploited indirectly through associated microorganisms (or the microbiome) that can hydrolyze DIP and make it available to their host^10,11^. The response of different microorganisms to DIP scarcity is likely distinct due to differences in the metabolic capabilities, and as a result has the potential to change microbial community structures. It is therefore crucial to understand the strategies used to circumvent DIP scarcity and to determine the importance of different P sources for microbial growth.

To assess the role of phosphorus for microbial growth, previous studies have performed radiolabeled (^32^P or ^33^P) phosphate and ATP uptake experiments, which provide important insights into the extent and mechanisms of DIP and DOP uptake at the single-cell level (e.g.^12,13^). Although microautoradiography combined with fluorescent *in situ* hybridization (MAR-FISH) links the identity of individual cells with DIP or DOP uptake, quantitative, cell-specific activities are commonly not reported (e.g.^14–17^) with some exceptions^18^. The combination of P radiotracers with cell flow-sorting has yielded quantitative insights into cellular P uptake rates for specific size classes/populations and phytoplankton taxa (e.g.^12,19,20^). While most studies have assessed DIP uptake, some have also used ^33^P-ATP as an organic P analogue. However, ATP can also be used as a carbon or nitrogen source^21^ and represents only a small fraction of the total organic P pool^22,23^. The DOP pool is diverse, and its concentration often exceeds that of DIP, suggesting that it is an important source of P in DIP-depleted environments^24^. The cell-specific alkaline phosphatase assay (e.g.^25–27^) has been used widely to study the use of organic P sources but is not a direct measure of uptake and cannot be easily linked to CO_2_ and N_2_ fixation rates in individual cells. Due to the complexity and the current methodological limitations, the quantitative importance of inorganic and organic P for growth of individual organisms in mixed environmental communities is largely unknown.

Here, we developed a new nanoSIMS-based, single-cell approach to directly quantify DIP uptake rates. These are measured simultaneously with C and N assimilation in the same cell and can therefore yield the contribution of DIP to the total P demand and growth of microorganisms. Meanwhile, measured single-cell elemental ratios allow for a mass balance to determine the importance of P storage and organic P utilization. Using this approach, we determined the quantitative importance of different P sources for three different harmful algal bloom (HAB) organisms in the Baltic Sea. The Baltic Sea suffers from seasonally recurring cyanobacterial HABs. These extensive blooms are usually dominated by one of three genera: (1) *Nodularia spumigena*, a producer of the toxin Nodularin^28^, (2) *Dolichospermum* sp., which in some cases can produce the toxin Microcystin^29^, and (3) *Aphanizomenon* sp., considered to be non-toxic^28^. But *Aphanizomenon*’s capacity to form dense blooms can contribute to, for example, oxygen depletion upon degradation and economic losses from reduced tourism (for review see^30^). In recent years, these noxious HABs have covered an area more than one-half of the entire Baltic Sea (~200,000 km^2^; ref.^31^). The HABs develop under conditions of seasonal shallow thermal stratification and are believed to be stimulated by excess DIP after the spring phytoplankton bloom has exhausted the dissolved inorganic nitrogen (DIN) pool^1^. Although the excess DIP is drawn down to nanomolar concentrations, the HABs continue to increase in size and intensity for several weeks^32^. It has been suggested that organic P^33^ as well as internal P storage^7^ are key factors in maintaining the HAB growth. While culture studies have suggested that *Nodularia* is superior to *Aphanizomenon* at using organic P^8,9^, the quantitative importance of these P sources in the environment is currently not known.

## Results and Discussion

### Single-cell approach

**Figure 1:**
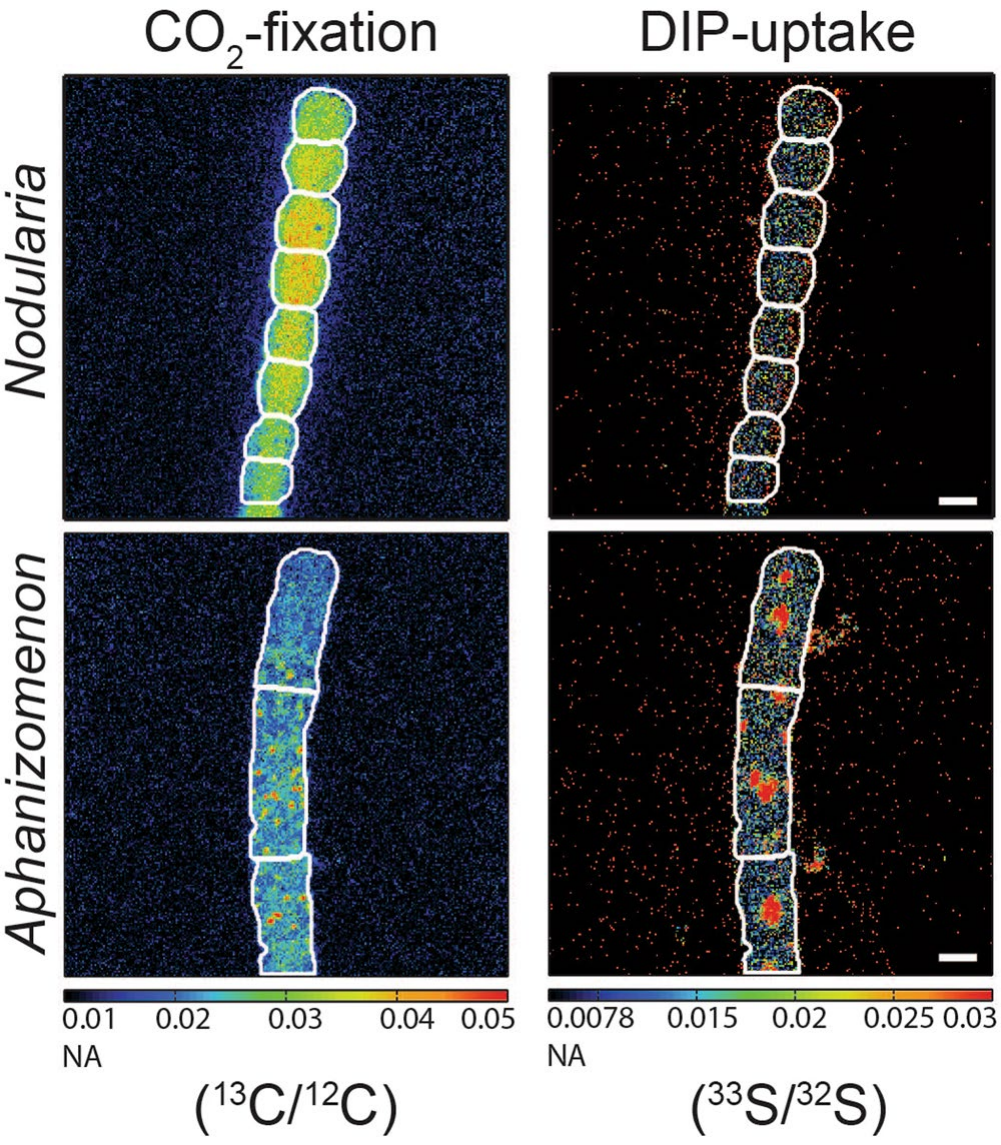
Single-cell imaging of cyanobacterial filaments. ^13^CO_2_ fixation and DI^33^P uptake as indicated by the ^13^C/^12^C and ^33^S/^32^S ratios measured via nanoSIMS in filaments of a *Nodularia* culture and filaments of *Aphanizomenon* collected in June from the Baltic Sea, both under sufficiently high DIP concentrations. White outlines indicate the cyanobacterial cells. NA = natural abundance. Scale bars are 3 µm for all images. An example of unenriched cells is given in Supplementary Fig. S2.

**Figure 2:**
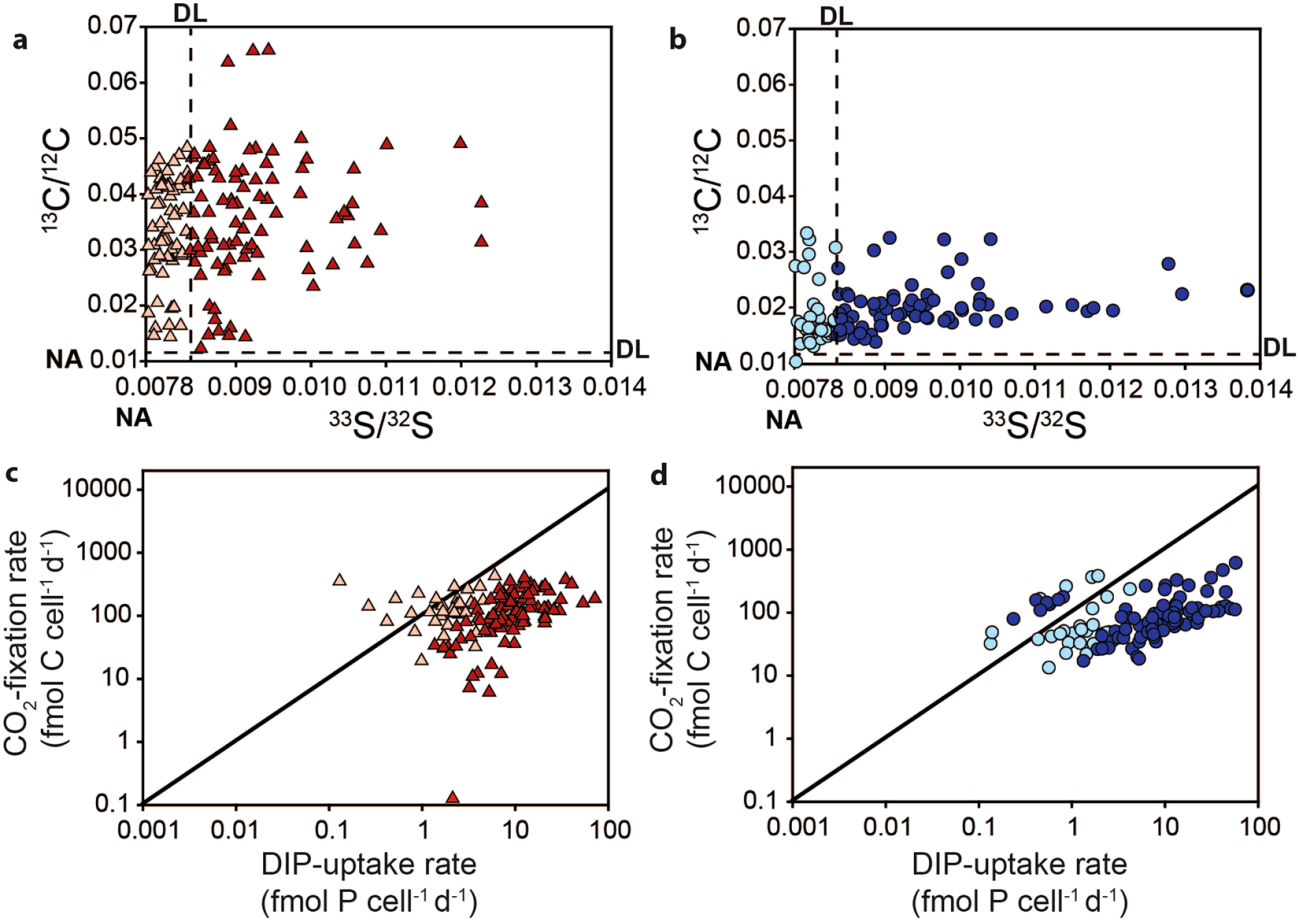
Single-cell isotopic enrichments and uptake rates of cyanobacteria. Cellular ^13^C/^12^C and ^33^S/^32^S enrichments by (**a**) a *Nodularia* culture and (**b**) *Aphanizomenon* collected in June from the Baltic Sea after incubations with ^13^CO_2_ and DI^33^P. The dashed lines indicate the detection limit of the isotopic enrichment, and cellular isotope ratios at or below the detection limit (DL) are shown in the faded colors. The CO_2_ fixation and DIP uptake rates for (**c**) the *Nodularia* culture and (**d**) *Aphanizomenon* were calculated from the isotopic enrichments (see methods) for all cells, with rates below the DL shown in faded colors. DL ^13^C/^12^C: 0.0117, DL ^33^S/^32^S: 0.0084. NA: natural abundance (0.01 for ^13^C/^12^C and 0.0078 for ^33^S/^32^S).

We developed a single-cell approach to measure the cellular DIP uptake rate using nanometer-scale secondary ion mass spectrometry (nanoSIMS). The measurement relies on the incorporation of radio-labeled (^33^P) phosphate into cellular biomass and the subsequent beta decay of the short-lived radioactive ^33^P (half-life ~25 d) to the stable ^33^S. The ^33^S/^32^S ratio is then used to calculate the DIP uptake rate (see methods). The challenge of this approach is to achieve high specific activities of ^33^P to attain enough ^33^P mass so as to significantly exceed the natural abundance of ^33^S (~0.75 atomic percent (at%)) in the cellular biomass (Supplementary Fig. S1; see methods). In addition to measuring a stable isotope, an advantage of measuring S isotopes instead of P isotopes is that sulfur is better ionized (~10-fold)^34^ by the nanoSIMS cesium (Cs^+^) primary ion beam resulting in increased ion counts and therefore improved statistics for determining the cellular DIP uptake rates. The measured cellular DIP uptake rate can be directly compared to cellular C and N assimilation rates of individual cells in mixed field populations when incubated with multiple isotopes in the same incubation. To test the developed method, we incubated a laboratory culture of *Nodularia spumigena* (KAC64) and field-collected colonies of *Aphanizomenon* sp. with ^13^CO_2_ and DI^33^P under DIP concentrations naturally occurring at the beginning of summer in the Baltic Sea (~150–300 nM DIP). The subsequent nanoSIMS analysis revealed the significant enrichment of both ^13^C and ^33^S isotopes in individual cells of the filamentous cyanobacteria indicating the cellular uptake and incorporation of CO_2_ and DIP into biomass (Fig. 1) with not all cells being enriched in ^33^S. This heterogeneity was observed for both the *Nodularia* culture and the field-collected *Aphanizomenon* and for both CO_2_ fixation and DIP uptake (Fig. 2a,b). Similar heterogeneity was previously reported for clonal^35–37^ and field-collected populations^38–40^ and was attributed to phenotypic heterogeneity.

Cell-specific rates were calculated for both cyanobacteria using the labeling percentage of ^13^C and ^33^P in the CO_2_ and DIP pool, the natural abundance of ^13^C and ^33^S, the incubation time and the cell size (Fig. 2c,d). The distribution of cellular rates is similar between the two cyanobacteria species with relatively high DIP uptake rates as compared to the canonical Redfield ratio suggesting that DIP concentrations were sufficient for both culture and field-collected colonies. As a proof of principle, these experiments demonstrate that cellular DIP uptake can be quantified using DI^33^P incorporation and subsequent nanoSIMS analysis of the cellular ^33^S/^32^S ratio simultaneously with carbon and/or nitrogen assimilation.

### Importance of P sources during harmful algal bloom

**Figure 3:**
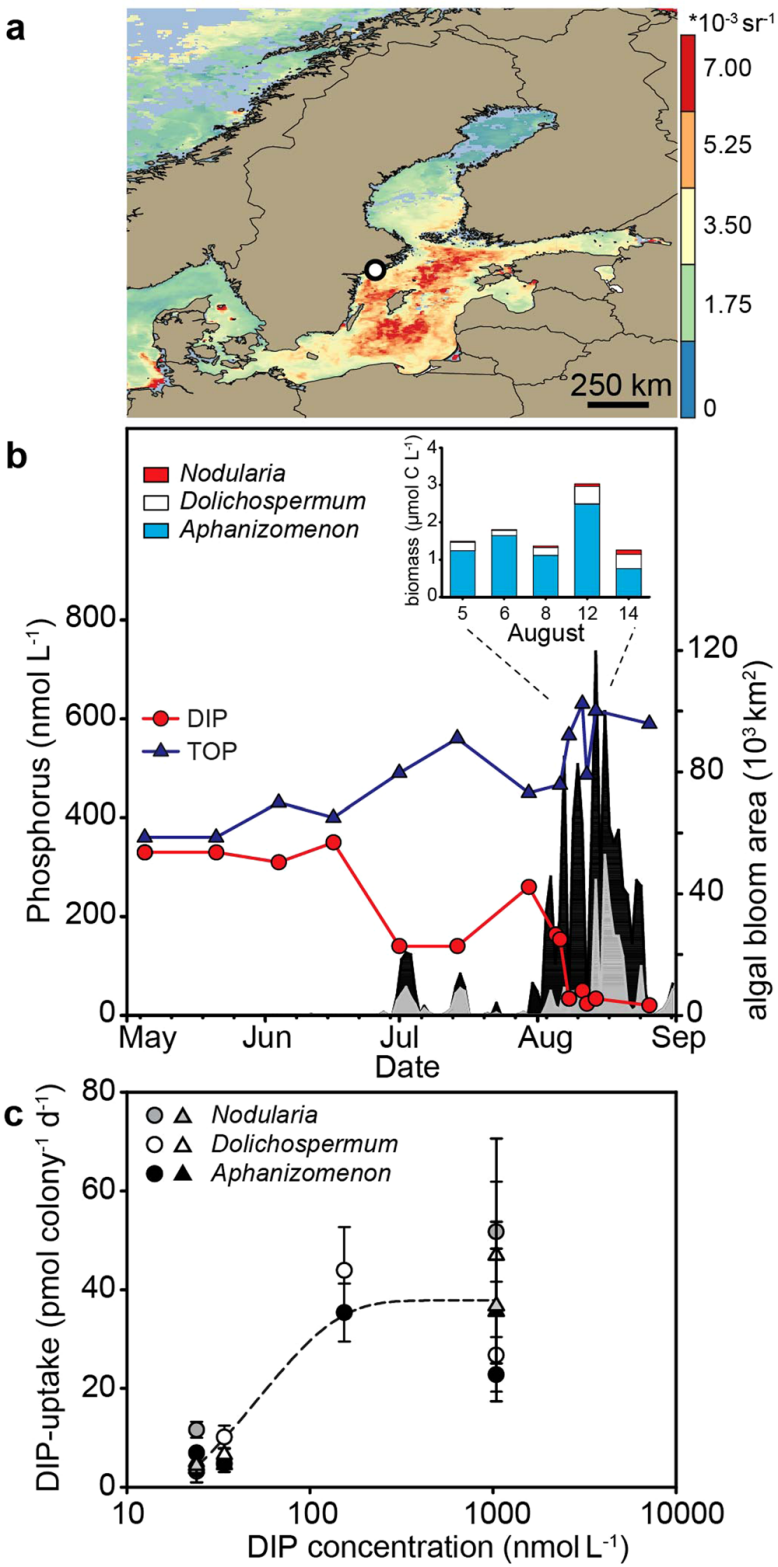
Harmful algal bloom (HAB) and DIP uptake rates in the Baltic Sea in summer 2015. (**a**) Composite satellite image (05–12 August) of cyanobacterial bloom (MODIS-Aqua; remote sensing reflectance at 555 nm), white dot: monitoring station B1. (**b**) DIP and total organic phosphorus (TOP) concentrations at station B1 (own data and open access data under the Creative Commons Attribution 4.0 at www.smhi.se), extent of the surface (black) and subsurface (white) bloom (data provided by^72^) and the cyanobacterial community structure during the sampling campaign; (**c**) colony-based DIP uptake rates during the bloom (circles = liquid scintillation counting, triangles = digital autoradiography). The dashed line shows the best fit which was used for the K_M_ determination.

We used the new method to determine the quantitative importance of DIP for the growth of three abundant HAB organisms at very low DIP concentrations in the summer. Our sampling took place during a cyanobacterial bloom that developed in early August 2015 when DIN was nearly depleted in the surface waters and the DIP concentration was ~250 nM (Fig. 3). The bloom covered large expanses of the Baltic Sea (Fig. 3a). At the Swedish National Monitoring Station B1 (Fig. 3a) the cyanobacterial bloom consisted mainly of *Aphanizomenon* sp., consistent with recurring summer blooms throughout the central Baltic^32,41^. *Dolichospermum* sp. and *Nodularia spumigena*, both (potential) toxin-producers, constituted about one to two orders of magnitude less biomass than *Aphanizomenon* sp., respectively (Fig. 3b). The development of the HAB caused a drawdown of DIP concentrations (Fig. 3b) from ~250 nM to 20–30 nM. This drawdown induced a strong decrease of the colony-based DIP uptake rates in the diazotrophic cyanobacteria from ~37 pmol colony^−1^ day^−1^ at ~150 nM DIP to ~7 pmol colony^−1^ d^−1^ at 34 nM DIP within 48 hours (Fig. 3c). Phosphate additions that raised the DIP concentration to >1 µM restored the uptake rates to those observed at a concentration of ~150 nM DIP. Logarithmic curve fitting (best fit) was used to determine the apparent half-saturation constant (K_M_ value) of ~60 nM DIP at very low DIP concentrations (Fig. 3c). This K_M_ value should be viewed cautiously, because these colonies also entrap organisms such as diatoms, flagellates and heterotrophic bacteria. DIP concentrations at the beginning of the bloom (~250 nM DIP) exceeded this apparent K_M_ value for phosphate uptake indicating that the onset of the bloom was not limited by DIP. In contrast, cyanobacteria became P-stressed after the onset and throughout the bloom, when concentrations had dropped to ~30 nM DIP (08 Aug 2015). Despite these very low DIP concentrations, the HAB increased in size and intensity (Fig. 3b).

**Figure 4:**
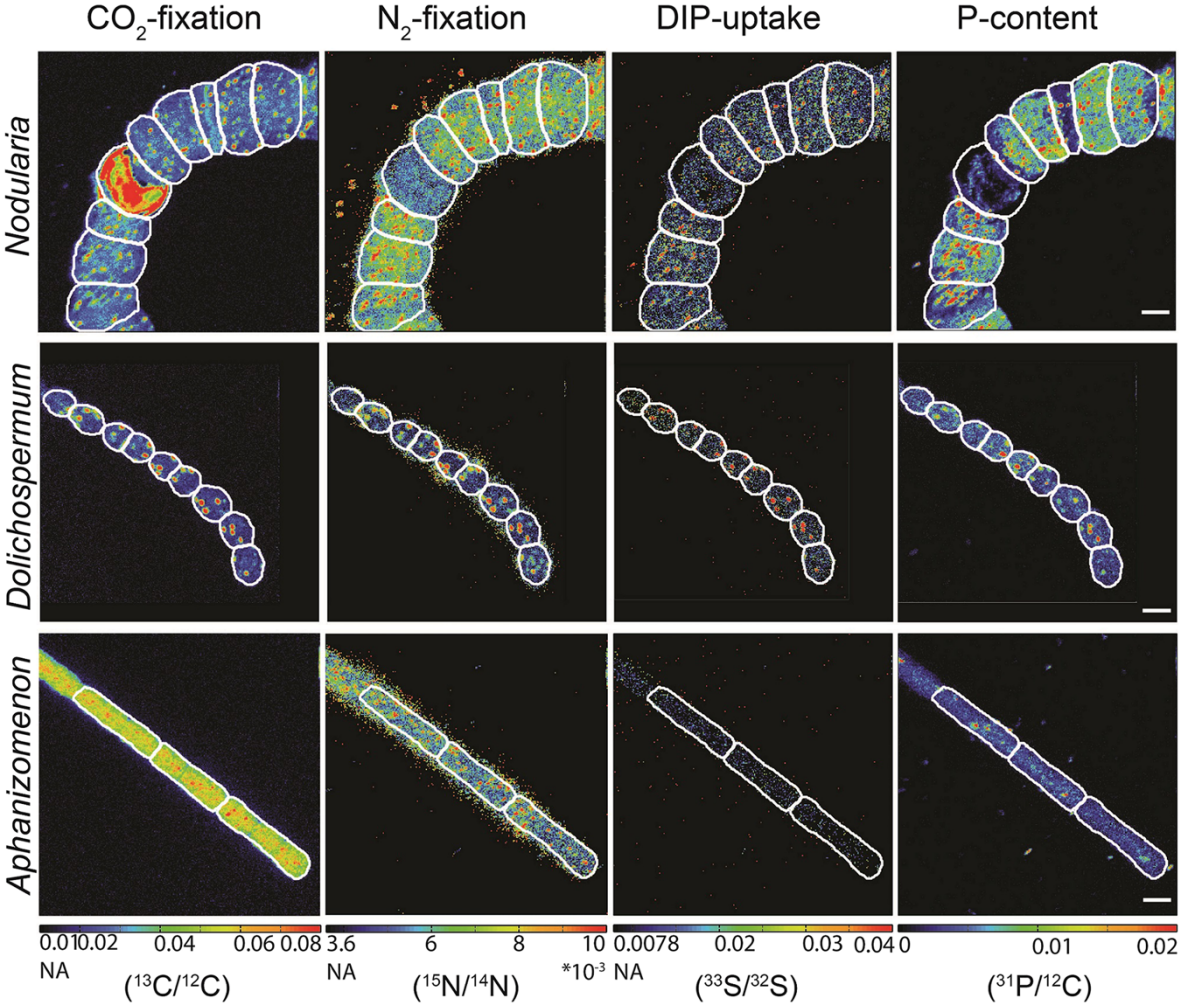
Single-cell imaging of cyanobacterial filaments collected during the cyanobacterial bloom in 2015. CO_2_ (^13^C/^12^C) and N_2_ (^15^N/^14^N) fixation and DIP (^33^S/^32^S) uptake measured through nanoSIMS for *Nodularia, Dolichospermum* and *Aphanizomenon*. The cellular P distribution is shown as the ^31^P/^12^C ratio. White outlines indicate the cyanobacterial cells. NA = natural abundance. Scale bars are 5 µm for all nanoSIMS images. *Dolichospermum* images have been size-adjusted to conserve the relative size proportions between the different cyanobacteria. For style reasons, these images have been framed with black bars.

**Figure 5:**
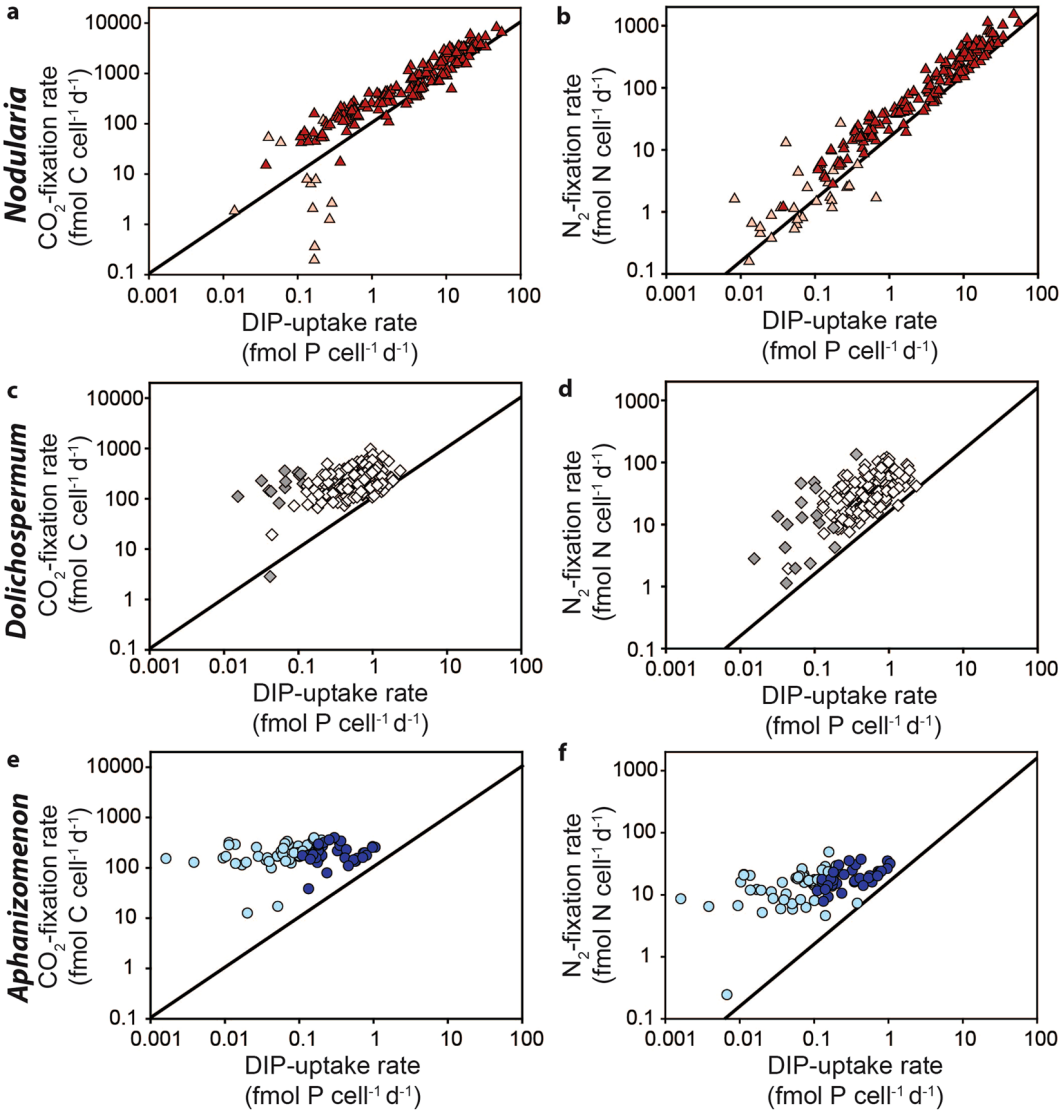
Single-cell uptake rates of cyanobacterial filaments. Single-cell CO_2_ and N_2_ fixation versus DIP uptake by *Nodularia* (**a**,**b**), *Dolichospermum* (**c**,**d**) and *Aphanizomenon* (**e**,**f**) under very low DIP concentrations. Solid lines indicate canonical Redfield ratios. Cellular rates at or below detection limit are indicated by faded colors (**a**,**b**,**e** and **f**) or by grey (**c**,**d**).

Single-cell nanoSIMS analyses of *Nodularia, Aphanizomenon* and *Dolichospermum* showed that all three cyanobacteria were actively fixing CO_2_ and N_2_ while also taking up DIP during the HAB (Figs 4 and 5). The measured CO_2_ and N_2_ fixation as well as the DIP uptake rates are likely conservative due to the use of fixatives during the sample processing^42,43^. Intriguingly, a fraction of the assimilated DIP appeared to be accumulating in granule-type areas of the cells despite DIP scarcity (Fig. 4). These granule-type areas were also hot-spots of freshly assimilated CO_2_ and N_2_ (Fig. 4). The exact nature of these granules is currently not known. Cyanophycin, a common storage product in cyanobacteria under P limitation^44^, itself does not contain phosphorus. However, freshly assimilated P is possibly stored as polyphosphates (polyP) in the same location as cyanophycin. PolyP formation and storage has traditionally been viewed as a ‘luxury’, where uptake occurs at DIP-replete conditions for use at times of P starvation^45^. However, more recent evidence suggests that polyP might play an important role for P cycling in phytoplankton during DIP scarcity^13,46,47^. Although the exact mechanisms are not known yet, it appears that phytoplankton conserve polyP during P starvation relative to other cellular P pools^47^. Nonetheless, the quantity of P stored in the observed granules is small (max. ~5%) here compared to the overall cellular DIP uptake.

Within each of the three populations of diazotrophic cyanobacteria, we observed considerable heterogeneity in C, N and P-uptake between cells, even within filaments (Supplementary Fig. S3), as has previously been observed for environmental populations^38–40^. In fact, the observed heterogeneity within a filament was often as large as the variability between filaments, and we statistically show that enough cells have been measured so that the results obtained here are representative for the different cyanobacteria (Supplementary Figs S4–S6). Most notable, however, was the difference in DIP uptake between the organisms. In *Nodularia*, CO_2_ and N_2_ fixation activity had a clear correlation to DIP uptake with ratios slightly higher than the Redfield ratio (Fig. 5a,b) while *Dolichospermum* showed a fairly weaker relationship of DIP uptake to CO_2_ and N_2_ fixation rates (Fig. 5c,d). Surprisingly, CO_2_ and N_2_ fixation had no relationship to DIP uptake in *Aphanizomenon* (Fig. 5e,f) indicating that P sources other than DIP were more important to its growth.

**Figure 6:**
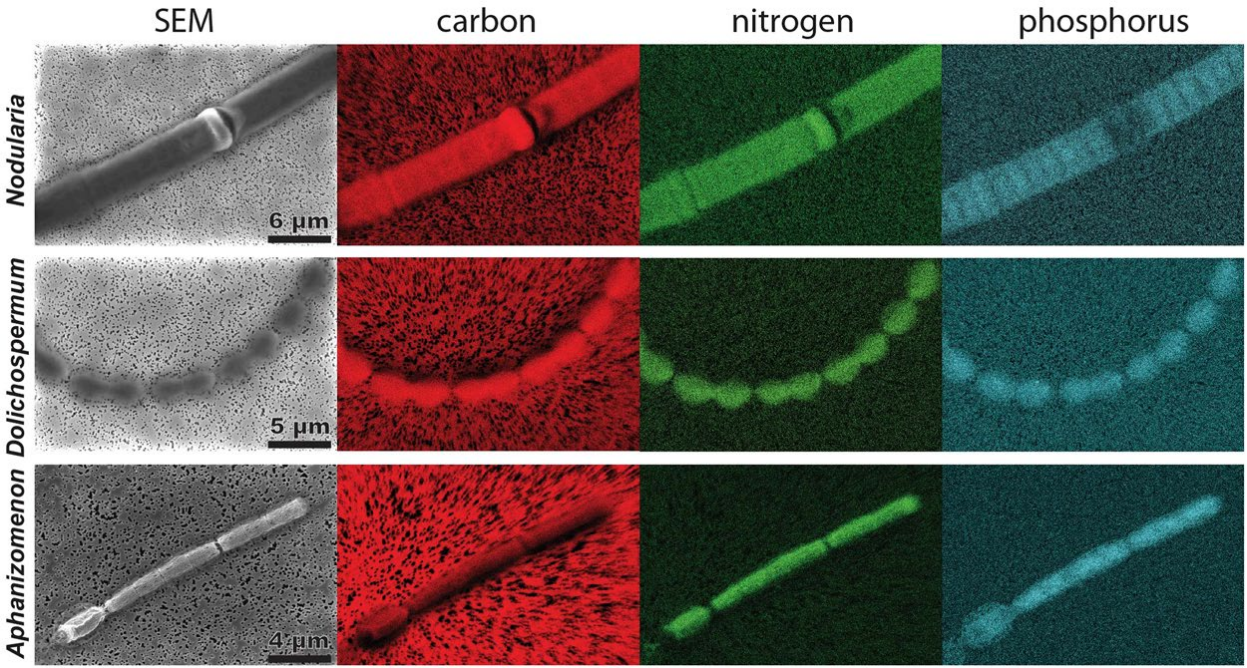
Single-cell elemental imaging of cyanobacterial filaments. Scanning electron microscopic (SEM) images of *Nodularia, Dolichospermum* and *Aphanizomenon* filaments with elemental imaging for carbon, nitrogen and phosphorus obtained via energy-dispersive X-ray spectroscopy (EDS). Scale bar size is indicated in the SEM image and is consistent within each of the cyanobacteria.

Baltic Sea filamentous cyanobacteria seem capable of adjusting their cellular elemental ratios to alleviate P stress^7,32^. A changing stoichiometry (C:P and/or N:P) could be the result of either using previously stored P or decreasing cellular P demand. To investigate whether P storage or a decreased cellular P demand could explain the difference between the organisms, we used energy-dispersive X-ray spectroscopy (EDS) to measure the elemental ratios of individual cells in colonies of all three cyanobacteria (Fig. 6).

The elemental ratios of all three cyanobacteria were already above Redfield at the beginning of our incubations indicating that P storage was not an important contributor to alleviating P stress, and therefore did not contribute to the increase in size and intensity of the cyanobacterial HAB in August 2015. However, we observed a shift in the elemental ratios within 24 hours. The C:P and N:P ratios of all three cyanobacteria increased by up to 45% and 200%, respectively (Supplementary Table S1) suggesting that the cellular P content decreased relative to C and N. While this changing stoichiometry likely contributed to alleviating or adjusting to P stress^7,32^, it cannot explain the differences between the cyanobacteria as the changes in the elemental ratios are similar for all three of them (Supplementary Table S1).

**Figure 7:**
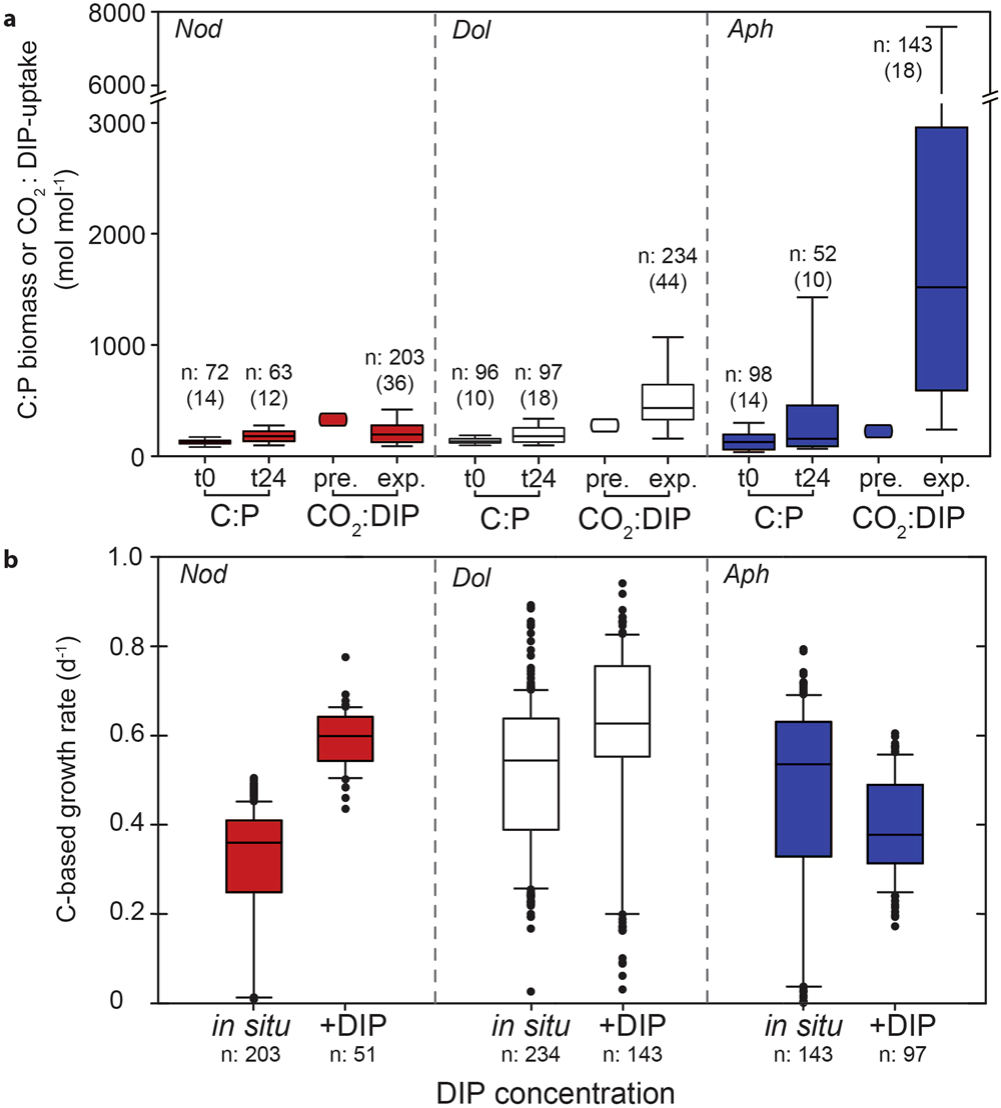
Cellular C:P ratios, CO_2_:DIP uptake ratios and C-based growth rates. (**a)** C:P biomass ratios in August at the start (t0) and end (t24) of the incubation (changes between these two time points were significantly different for all three cyanobacteria, Kruskal Wallis test p < 0.05) and the predicted (pre.) and experimentally (exp.) measured CO_2_:DIP-uptake ratios; lines = medians, boxes = 25^th^ and 75^th^ percentiles, bars = standard deviation. Outliers (indicated by numbers in parentheses below number of measured cells (n)) are not plotted here for easier visualization but were all included in the analysis. (**b)** Growth rates based on ^13^CO_2_ incorporation at *in situ* and added DIP (+1 µM) concentrations; lines = medians, boxes = 25^th^ and 75^th^ percentiles, bars = standard deviation, black circles = outliers). All changes in growth rates between the two different DIP concentrations were significant for all three cyanobacteria, Kruskal Wallis test, p < 0.05. Numbers in parentheses below X-axis indicate the number of measured cells. (**a**,**b)** *Nodularia* (red, *Nod*), *Dolichospermum* (white, *Dol*) *and Aphanizomenon* (blue, *Aph*). Cellular elemental ratios at t0 and t24 can also be found in Supplementary Table S1.

Apart from DIP, organic P compounds can serve as P sources for cyanobacterial growth. Since the organic P pool is very diverse, it is nearly impossible to measure the direct uptake of total organic P. Nevertheless, if the total P demand for cellular growth is compared with the measured DIP uptake, the contribution of total organic P can be calculated. We calculated the mass balance of total P demand for cellular growth using the C-based growth rates (from ^13^CO_2_ assimilation) and the changing elemental stoichiometries over the incubation time (see Methods). This mass balance predicts the CO_2_:DIP uptake ratio that would be necessary to sustain all growth by DIP (Fig. 7a). The predicted CO_2_:DIP uptake ratio is then compared to the experimental CO_2_:DIP uptake ratio obtained from the nanoSIMS analysis to reveal the importance of DIP and organic P. If the ratios are similar, (nearly) all growth can be sustained by the experimentally measured DIP uptake. However, if the experimental ratio is higher than the predicted ratio (i.e. less DIP uptake relative to carbon was measured), not all growth can be sustained by DIP. Since the changing elemental composition has already been taken into account at this point (including potential storage), the difference between the predicted and the experimental P uptake must be satisfied by organic P sources.

The predicted CO_2_:DIP uptake ratio was very similar to the experimental, isotope-based ratio of CO_2_ fixation to DIP uptake for *Nodularia* (Fig. 7a) indicating that this cyanobacterium relied almost entirely on DIP uptake for its growth, even at very low DIP concentrations. In contrast, a massive mismatch was observed between the predicted and the experimental ratio for *Aphanizomenon* (Fig. 7a) indicating substantial utilization of organic P: approximately 85% of its cellular P was obtained from organic P while only 15% was acquired from DIP uptake. *Dolichospermum* showed an intermediate strategy (Fig. 7) with about 40% of phosphorus originating from organic P and 60% from DIP. This segregation of phosphorus usage in field populations is surprising as cultured isolates of all three cyanobacteria are (genetically) capable of using organic P^48–51^. Further, this result from the field is at odds with several previous laboratory-based, culture studies suggesting that *Nodularia* outcompetes *Aphanizomenon* for organic P sources^8,9^. One mechanism to increase an organism’s competitiveness is to make use of the surrounding microbiome (or phycosphere). Interactions between a ‘host’ and its microbiome include but are not limited to mutualism and competition^52^. Associated microorganisms of the filamentous, non-heterocystous cyanobacterium *Trichodesmium*, for example, possess an array of tools for the hydrolysis of DIP from organic P through alkaline phosphatases which could then potentially be taken up by *Trichodesmium* itself^11^. The cyanobacterium *Aphanizomenon ovalisporum* uses the production of the toxin cylindrospermopsin to induce extracellular alkaline phosphatase activity in its surrroundings^53^. The thereby cleaved DIP could then be used by *A. ovalisporum* to satisfy its P demand. Alkaline phosphatases cleave the DIP (i.e. phosphate or P_i_) from phosphoester bonds, and can exist as extracellular, membrane-bound, periplasmic, or cytoplasmic enzymes, although the majority appears to be extracellular and cytoplasmic in marine bacteria^54^. In our experiments, the DIP assimilated by *Nodularia* (as measured by the ^33^P incorporation) can originate from the initial ^33^P-labeled DIP pool (Supplementary Fig. S7; pathway (1) as well as from non-labeled DIP that has been released from organic P by extracellular hydrolysis by *Nodularia* or hydrolysis by surrounding microorganisms. Only the direct uptake from the initial ^33^P-labeled DIP pool will be captured by our ^33^P-DIP method. Since the measured ^33^P-labeled DIP uptake by *Nodularia* almost entirely accounts for their cellular P demand, it is very unlikely that they used a substantial amount of organic P (Supplementary Fig. S7; pathway 2–4). The indirect use of DOP by *Aphanizomenon* (pathways 3 and 4 in Supplementary Fig. S7) cannot be completely excluded here but is unlikely given the sparse epibiotic population of *Aphanizomenon* filaments^38,55^.

In essence, our field study shows that internally stored P did not play a substantial role in extending the HAB through late summer as elemental ratios were already around Redfield at the start of the bloom. The plasticity in the cellular elemental stoichiometry likely contributed to the growth of all three cyanobacteria with elemental ratios exceeding Redfield already a few days after the onset of the bloom. Most remarkably, however, organic P is used unequally by the three cyanobacteria with *Nodularia* relying almost exclusively on DIP and *Aphanizomenon* retrieving the majority of its cellular P from organic P. When DIP was added to incubations, *Aphanizomenon* did not grow faster than at very low DIP concentrations (Fig. 7b) suggesting that *Aphanizomenon*’s growth is independent of the availability of DIP during late summer. In contrast, the growth rate of *Nodularia* was stimulated when DIP was added to our incubations from 0.35 to 0.59 d^−1^ (Mann-Whitney test, p < 0.01) (Fig. 7b). Interestingly, *Dolichospermum* showed only a slightly enhanced growth rate resembling its intermediate DIP usage (Fig. 7). Overall our results indicate that the different strategies used to overcome summertime DIP scarcity are mostly based on differences in organic P usage between the different cyanobacteria. While several factors control the spatial and temporal distribution of the different cyanobacteria in the Baltic Sea, we hypothesize that its efficiency to use organic P leads to the numerical dominance of *Aphanizomenon* in the central Baltic during the stratified summer period when the three cyanobacteria co-occur (Fig. 3b and^56^). In contrast, the growth of *Nodularia* appears to benefit from the availability of DIP. A modelling study found that the best fit to observations of *Nodularia* blooms in the Baltic Sea could only be explained if DIP input by turbulent mixing and upwelling events were considered in addition to excess DIP in the water at the start of summer^57^. Further, a mesocosm study in the Baltic Sea found that growth of *Nodularia* was enhanced relative to *Aphanizomenon* and *Dolichospermum* when DIP was added to the water^58^. Both studies are in agreement with our results and together suggest that occurrences and abundances of *Nodularia* could largely benefit from increased DIP availability.

Modelling projections of the environmental conditions in the Baltic Sea under changing climate and nutrient inputs in the 21^st^ century reveal that most scenarios (including business-as-usual) will lead to increased nutrient availability in surface waters, in particular phosphorus^59^. Furthermore, a major fraction of the phosphorus supply to the Baltic Sea is from deeper waters that accumulate DIP released from anoxic/hypoxic sediments^60^. Increases in anoxia and hypoxia have thus kept P quota in the Baltic Sea at the same or increased levels even when inputs have been reduced^60^. Our results imply that when the overall DIP availability increases and/or DIP is introduced to surface waters during summer stratification (e.g., by turbulent mixing, upwelling or point sources), out of the three cyanobacteria, the toxin-producer *Nodularia* would benefit most and potentially outcompete other HAB organisms. An increased availability or external input of DIP could thus trigger or increase blooms of *Nodularia* and shift non-toxic blooms toward more toxic ones by changing the community structure. The extent, intensity and frequency of HABs worldwide are predicted to increase under global climate change^61^ and eutrophication via anthropogenic activity^62^. The capability of HAB organisms to use organic P may be a critical factor that not only determines the microbial community structure, but the overall harmfulness and associated costs of algal blooms.

Our new approach allows for the simultaneous measurement of cellular DIP uptake and C and N assimilation rates in individual cells. The combination of these rates with the cellular elemental stoichiometry enabled us to quantify the importance of DIP, P storage and organic P for the growth of harmful cyanobacteria in a mixed environmental community. This approach could be applied in other low-phosphate ecosystems to determine the importance of different P sources for microbial growth and the potential role of organic P in the success of bloom-forming organisms.

## Methods

### Sampling and experimental set-up

#### Culture experiments

*Nodularia spumigena* (KAC64) cultures were grown on artificial seawater medium (BG-11) adjusted to a salinity of 7 and without the addition of fixed N. Growth conditions were set to room temperature (~20 °C) and a 12:12 h light/dark cycle. To determine single-cell CO_2_ and DIP-uptake rates cultures were transferred to fresh medium (150 nmol DIP L^−1^) 48 hours prior to the incubation experiment. For the incubation experiments, medium was enriched with NaH^13^CO_3_ (≥98 atom%, Sigma-Aldrich), resulting in a final enrichment of 13.6 atom% ^13^C in the DIC pool, and transferred carefully to 6-mL Exetainers. To each of these Exetainers a single hand-picked *Nodularia* colony was added. Afterwards, ~3 MBq ^33^PO_4_^3−^, corresponding to 1.57 pmol ^33^P, were added to each Exetainer. This resulted in an isotope enrichment of 0.16 atom% ^33^P (at 150 nmol DIP L^−1^) with no significant natural background of ^33^P. Incubations lasted 24 hours and were terminated and prepared for further analysis as described below.

#### Field experiments

In June 2015, field sampling took place at the Swedish National Monitoring site B1 (5848′18″N, 17°37′52″E) close to the south-eastern coast of Sweden using a small motor-driven boat. Bulk water and *Aphanizomenon* colonies (the only abundant cyanobacterium) were collected with 30-L high-density polyethylene (HDPE) containers (Nalgene) and a plankton net (mesh size 20 μm), respectively, on 13 June 2015. Samples were transported back to the Askö Laboratory (Baltic Sea Center) within 30 minutes after sampling. Bulk seawater was subsampled for nutrient concentrations while *Aphanizomenon* colonies were incubated with H^13^CO_3_^−^, ^15^N_2_ and ^33^PO_4_^3−^ to measure single-cell CO_2_, N_2_ and DIP assimilation rates, respectively, using the developed method (see ‘Single-cell CO_2_, N_2_ and DIP assimilation’ below). Incubations were carried out in a temperature- and light-controlled room that was set for *in situ* conditions measured on the sampling day at station B1 at a 1–2 m water depth (11.7 °C; 280 µmol photons m^−2^ s^−1^ light irradiance; 18 h:6 h light:dark cycle).

In August 2015, field sampling was carried out as described above on three different occasions (06, 08 and 12 August 2015) at the same site. Samples were transported back to the Askö Laboratory (Baltic Sea Center) within 30 minutes after sampling.

In the first experiment, surface water was subsampled for nutrient (NH_4_^+^, NO_2_^−^/NO_3_^−^ and PO_4_^3−^) concentrations and the abundance of filamentous cyanobacteria, while the colonies were used to determine colony-based DIP uptake rates using the radiotracer ^33^P. The second experiment was carried out as described above with the addition of incubations for single-cell rates of CO_2_ fixation, N_2_ fixation and DIP uptake using a triple isotope approach with H^13^CO_3_^−^, ^15^N_2_ and ^33^PO_4_^3−^, respectively. A third experiment was carried out as described for the first experiment with additional colony incubations under artificially increased (+1 µmol L^−1^) DIP concentrations and with the addition of stable isotopes H^13^CO_3_^−^ and ^15^N_2_. All incubations were carried out in a temperature- and light-controlled room that was set for *in situ* conditions measured on the sampling day at station B1 at a 1–2 m water depth (17.4–18.0 °C; ~280 µmol photons m^−2^ s^−1^ light irradiance; 18 h:6 h light:dark cycle).

### Nutrient concentrations

Samples for ammonium (NH_4_^+^) concentration were taken immediately upon arrival at the laboratory and subsequently measured fluorometrically^63^. Subsamples for phosphate (PO_4_^3−^; dissolved inorganic phosphorus, DIP) and nitrite/nitrate (NO_2_^−^/NO_3_^−^) concentrations were pre-filtered (0.45 µm pore size Millex® syringe filter; Millipore) and frozen at −20 °C until analysis while samples for total phosphorus (TP) were frozen untreated at −20 °C. DIP concentration was measured with an auto-analyzer (TRAACS 800 Bran Luebbe) equipped with a 1-m long capillary system^64^ (World Precision Instruments) at the Max-Planck-Institute for Marine Microbiology within three months of sampling. Both nitrite/nitrate (NO_2_^−^/NO_3_^−^) and total phosphorus (TP) concentrations were determined by the Stockholm University Baltic Sea Monitoring Group using a standard flow injection analysis (FIA, QuikChem 8000 method: 31-107-06-1-A Lachat Instruments), and oxidation of phosphorus by pyrosulfate coupled to a segmented flow system^7^ (ALPKEM Flow Solution IV). The total organic phosphorus concentration (TOP) was then calculated by subtracting DIP from the TP concentrations. Nutrient concentrations at station B1 before and after our sampling campaign were retrieved from the Swedish Meteorological and Hydrological Institute (SMHI) database at www.smhi.se, open access data under the Creative Commons Attribution 4.0.

### Abundance of filamentous cyanobacteria

*Aphanizomenon* sp., *Dolichospermum* sp. and *Nodularia spumigena*, were identified and counted in (acid) Lugol-preserved seawater as described in the HELCOM Guidelines^65^.

### Colony-based DIP-uptake rates

Cyanobacterial colonies were identified using a stereomicroscope, hand-picked with a needle, washed in filtered seawater (0.2 μm pore size) and transferred to 6 mL gas-tight Exetainer^®^ filled with filtered seawater. The colony-based DIP uptake rates were measured using radiolabeled phosphate (150 kBq ^33^PO_4_^3−^ in 6 mL of filtered (0.2 µm) water, specific activity 3000 Ci mmol^−1^, t_1/2_ 25.4 d; Hartmann Analytic) in two different sets of incubations: for the first set, each Exetainer^®^ contained ten colonies for rate measurements using liquid scintillation counting (LSC), for the second set each Exetainer^®^ contained a single colony for rate measurements using digital autoradiography (DAR) analysis. Each of these two sets contained triplicate Exetainer^®^ incubations per time point (0, 6, 12, 18 and 24 h). At each time point, the triplicate sets were filtered through polycarbonate filters (GTTP, 0.2 µm pore size, 25 mm diameter, Millipore) and washed twice with 5 mL of filtered-seawater for LSC The filters were then transferred to a 6 mL scintillation vial containing 5 mL scintillation fluid (Irga-safe plus, PerkinElmer). For DAR analysis, the samples were preserved with formaldehyde (1% w/v final concentration) for 30 minutes at room temperature prior to filtering. DAR samples were mounted onto microscope glass slides, using a small drop of 0.1% low-melting-point agarose, dried and stored at room temperature before analysis using a real-time radio imaging system (Micro Imager, BioSpace Lab) as described in Goldhammer *et al*.^66^.

Total radioactivity was determined from every incubation bottle using 100 µL of water that were directly transferred into a scintillation vial. All samples from one experiment were measured on the same day using a liquid scintillation counter (type: 425-034, Hidex). Control incubations were carried out with heat-killed (80 °C, 30 minutes) or no colonies. Both controls were used to check for any unspecific binding of the radiotracer to the bottle, filter or organic material. Colony-based DIP uptake rates were calculated using the linear regression for the radiotracer incorporation into biomass between time points, the total amount of radiotracer added and the DIP concentration in the water. Uptake rates were corrected for radiotracer decay, and unspecific radiotracer binding using the controls. Uptake rates were normalized to the number of colonies per incubation.

### Single-cell CO_2_, N_2_ and DIP assimilation

Single-cell CO_2_, N_2_ and DIP-uptake rates were determined using a triple isotope approach followed by nanometer-scale secondary ion mass spectrometry (nanoSIMS). In detail, a 2.5-L polycarbonate bottle with filtered seawater (0.2 μm pore size) was enriched with NaH^13^CO_3_ (≥98 atom%, Sigma-Aldrich) and ^15^N_2_ (≥99 atom%; lot # 1-17299, Cambridge Isotope Laboratories; modified bubble method as in Klawonn *et al*.^67^). The prepared water was carefully transferred to 6-mL Exetainers to which a single hand-picked cyanobacterial colony was added. Subsequently, ~10 MBq ^33^PO_4_^3−^, corresponding to 4.95 pmol ^33^P, were added to each Exetainer. This resulted in an isotope enrichment of 0.28 atom% ^33^P (294 nmol DIP L^−1^) in June and 2.43 atom% in August (34 nmol DIP L^−1^), with no significant natural background of ^33^P. These ^33^P enrichments led to detection limits of ~0.107 and 0.012 of relative P incorporation for June and August, respectively (Supplementary Fig. S1). After the addition of ^33^PO_4_^3−^, the Exetainers were closed headspace-free and incubated as described above. Per cyanobacterial genera three samples were terminated after 24 hours (t24) for June and August and a single sample was terminated at time zero (t0) (i.e. immediately after the last tracer was added) for August only. One of the three sampled colonies was later used for SEM-EDS analysis while the other colonies were used for nanoSIMS measurements. Total radioactivity was determined from each Exetainer as described above except that the 100 µL of incubation water were first diluted ten-fold with nanopure water and 100 µL from this dilution were used for the measurement. The remaining sample was fixed with formaldehyde (1% w/v final concentration) for 30 minutes at room temperature and then filtered through a gold-sputtered polycarbonate filter (0.22 μm pore size; 25 mm diameter; Millipore). The filters were washed with nanopure water, dried at room temperature and mounted onto microscope glass slides using a small drop of 0.1% low-melting-point agarose. Microscope slides were stored in the dark at room temperature before single-cell analyses.

#### Single-cell elemental ratios

Single-cell C:N, C:P and N:P elemental ratios were obtained in August samples using a scanning electron microscope (SEM; Quanta FEG 250, FEI) equipped with an energy-dispersive X-ray spectroscopy (EDS) double detector system (QUANTAX EDS, Bruker Nano GmbH) at the Max Planck Institute for Marine Microbiology. The detectors (XFlash 6/30) had a detector area of 30 mm^2^ and an energy resolution at Mn K α line of <123 eV, which allows for quantification of light elements. The NBS SRM 1155 ANSI 316 stainless steel standard was used to check the performance of the EDS system. Prior to EDS measurements, we tested the EDS spectra with an accelerating voltage of 10 kV and 20 kV. The same elements up to Ca could be detected with both voltages, sufficient to obtain ratios for the light elements. In order to reduce the penetration depth, and thereby reduce potential signals from the filter, the 10 kV accelerating voltage was used for further measurements. Monte-Carlo simulations showed that a 10 kV accelerating voltage led to a penetration depth of ~2 µm indicating that the obtained elemental ratios were representative for the cellular composition due to minimal interference from the filter. The analysis of the cellular elemental content was done using the P/B-ZAF method (Quantax 400 software, version 1.9; Bruker) in which the elements C, N, O, Na, Mg, Al, P, S, Cl, K and Ca were included. This analysis relies on the ratio of the X-ray intensities relative to the intensities of the Bremsstrahlung, which is simultaneously recorded by the EDS detector. The P/B-ZAF method is a standardless evaluation without the need for normalization and is particularly useful for the analysis of rough surfaces and particles. Analyses were done from samples of the beginning and the end of the incubation for all three different cyanobacteria in August. Significant differences in the elemental ratios were tested using a Kruskal-Wallis test (for non-normal distribution) (Fig. 7 and Supplementary Table S1).

#### nanoSIMS analysis

Single-cell CO_2_, N_2_ and DIP uptake rates were determined based on the incorporation of DI^13^C, ^15^N_2_ and ^33^PO_4_^3−^ into the cyanobacteria cell biomass. The radiotracer ^33^P decays to the stable ^33^S with a half-life of only 25.4 days and becomes part of the cellular sulfur pool. Therefore, DIP-uptake rates were measured based on the labelling excess of ^33^S/^32^S (natural abundance ratio of 0.0078) in the biomass using a nanoSIMS 50 L (CAMECA) at the Max Planck Institute for Marine Microbiology in Bremen, Germany. Measurements were done after at least four half-lives had passed to ensure that the majority of the ^33^P had decayed.

*Aphanizomenon*, *Dolichospermum* or *Nodularia* filaments were identified based on auto-fluorescence and morphology and were marked using a laser micro-dissection (LMD) microscope (6000B, Leica). After loading the sample into the nanoSIMS, marked filaments were pre-sputtered with the Cs^+^ primary ion beam of 300 pA to remove surface contamination, implant Cs^+^ ions into the sample and to achieve an approximately stable ion emission rate. A primary Cs^+^ ion beam with a beam current between 1 and 1.2 pA and a beam diameter between 50 and 100 nm was rastered across the cells for analysis. For individual cells, secondary ion images of ^12^C^−^, ^13^C^−^, ^12^C^14^N^−^, ^12^C^15^N^−^, ^31^P^−^, ^32^S^−^ and ^33^S^−^ were simultaneously recorded using seven electron multipliers. The analysis areas were between 20 × 20 and 50 × 50 µm in size and an image size of 256 × 256 pixels with a dwell time of 1 ms per pixel and a minimum of 50 planes. To minimize interferences for ^12^C^15^N^−^ and ^33^S^−^ the instrument was tuned for high mass resolution (>7000 MRP). All nanoSIMS measurements were analyzed using the Matlab-based software package Look@NanoSims^68^ (available at http://nanosims.geo.uu.nl/nanosims-wiki/doku.php/nanosims:lans). For every measurement, secondary ion images were drift-corrected and accumulated. Regions of interest (ROIs) were drawn around cells using the secondary ion images, and ^13^C/^12^C, ^15^N/^14^N (inferred from ^12^C^15^N/^12^C^14^N), ^33^S/^32^S and ^31^P/^12^C ratios were calculated.

Accuracy and precision of the ^33^S/^32^S measurements were determined with an in-house sulfate standard (BaSO_4_) which had natural abundance values. Cells were regarded as significantly enriched when their ^33^S/^32^S ratio was above the natural abundance +3 × standard deviation of the mean of the sulfate standard, i.e. a ^33^S/^32^S ratio of 0.0084 was the detection limit with a natural abundance value of 0.0078.

#### Single-cell CO_2_, N_2_ and DIP uptake rates

Individual cell biovolumes were calculated based on cell-diameter measurements, obtained via nanoSIMS analysis, in combination with a geometrical shape model. For *Aphanizomenon* and *Dolichospermum* we used a cylinder +2 half spheres-shape and for *Nodularia* a cylinder-shape^69^. Cellular C content was determined based on the biovolume and a volume-to-carbon conversion factor of 17.5 fmol C μm^−3^ (ref.^70^). Cellular N and P content was calculated using the molar C:N and C:P ratios obtained via SEM-EDS described above.

CO_2_ and N_2_ fixation rates were calculated using the cellular C and N content in combination with the single-cell ^15^N_2_ and DI^13^C incorporation, analogous to the calculations described in Musat *et al*.^71^. Single-cell DIP-uptake rates were calculated using the cellular P-content (P_cell_) obtained via the SEM-EDS, DI^33^P incorporated in the cell measured as ^33^S excess (^33^S_excess_), the DI^33^P labelling yield (^33^P_excess-inc_) and incubation times (t) as follows:

1 $$\mathrm{DIP} \mbox{-} \mathrm{uptake}\,{\rm{rate}}=({}^{33}{\rm{S}}_{{\rm{excess}}}/{}^{33}{\rm{P}}_{\mathrm{excess} \mbox{-} \mathrm{inc}})\times {{\rm{P}}}_{{\rm{cell}}}\times (1/{\rm{t}})$$

#### Growth rates

Growth rates were determined based on the incorporation of DI^13^C (C_excess_) into the biomass assuming an even distribution of the isotope in the biomass during cell division as follows:

2 $$C \mbox{-} \mathrm{based}\,{\rm{growth}}\,{\rm{rate}}\,[{{\rm{d}}}^{-1}]=({{\rm{C}}}_{{\rm{excess}}}/{{\rm{C}}}_{\mathrm{excess} \mbox{-} \mathrm{inc}})\times 2\times 1/{\rm{t}}$$

with C_excess-inc_ representing atom%-labelling of DIC in the bottle and t the incubation time. A Kruskal-Wallis test was used to determine significant differences in C-based growth rates between species and incubation conditions (*in situ* or added DIP).

### Contribution of DIP and organic P to cellular growth

To determine the contribution of DIP to cellular growth, we compared the experimental CO_2_:DIP uptake ratio, obtained from the nanoSIMS analysis, with the predicted ratio of CO_2_:DIP (CO_2_:P). The latter was determined from the mass-balanced cellular growth between the beginning and the end of the incubation as follows:

3 $${{\rm{M}}}_{{\rm{final}}}\times {{\rm{R}}}_{{\rm{final}}}={{\rm{M}}}_{{\rm{start}}}\times {{\rm{R}}}_{{\rm{start}}}+{{\rm{M}}}_{{\rm{new}}}\times {{\rm{R}}}_{{\rm{uptake}}}$$

which is then solved for R_uptake_, i.e. the predicted ratio

4 $${{\rm{R}}}_{{\rm{uptake}}}=({{\rm{M}}}_{{\rm{final}}}\times {{\rm{R}}}_{{\rm{final}}}-{{\rm{M}}}_{{\rm{start}}}\times {{\rm{R}}}_{{\rm{start}}})/{{\rm{M}}}_{{\rm{new}}}$$

with M = median biomass at the start (M_start_; obtained from cell size and volume conversion) or end (M_final_; calculated by growth rates of start biomass) of the incubation and the newly formed biomass (M_new_; difference between start and end biomass) during the incubation and with R = median elemental ratio of the biomass at the start (R_start_) and end (R_final_) of the incubation (both obtained from the SEM-EDS) and the median uptake ratio of CO_2_:DIP (R_uptake_) during the incubation.

The contribution of DIP and organic P uptake to cellular growth was then calculated by dividing the ratio of uptake rates obtained by nanoSIMS (CO_2_:DIP) by the predicted ratio (CO_2_:P, i.e. R_uptake_). To obtain the fraction of DIP supporting cellular growth the resulting number (P/DIP) was inverted (DIP/P). It follows that the cellular growth supported by organic P = (1 − DIP/P) with DIP/P + organicP/P = 1.

## Electronic supplementary material

Supplementary Information
