# Supplementary material for: Single-cell imaging of phosphorus uptake shows that key harmful algae rely on different phosphorus sources for growth
Source: Sci Rep. 2018 Nov 21;8:17182. doi: 10.1038/s41598-018-35310-w (PMC6249326; doi:10.1038/s41598-018-35310-w)
Supplement: Supplementary file 1 — Supplementary Information [file 41598_2018_35310_MOESM1_ESM.pdf]

Supplementary Information

Single-cell imaging of phosphorus uptake shows that key harmful algae rely on different phosphorus sources for growth

Niels J. Schoffelen [1], Wiebke Mohr [1*], Timothy G. Ferdelman [1], Sten Littmann [1], Julia Duerschlag [1], Mikhail V. Zubkov [2], Helle Ploug [3], Marcel M.M. Kuypers [1]

Affiliations:

[1] Department of Biogeochemistry, Max Planck Institute for Marine Microbiology, Celsiusstraße 1, 28359 Bremen, Germany, [2] Ocean Biogeochemistry and Ecosystems, National Oceanography Centre Southampton, European Way, Southampton, SO14 3ZH, United Kingdom, [3] Department of Marine Sciences, University of Gothenburg, Carl Skottsbergs Gata 22B, 41319 Gothenburg, Sweden

[*] For correspondence: Wiebke Mohr, Max Planck Institute for Marine Microbiology, Celsiusstraße 1, 28359 Bremen, Germany, Phone: +49 (0)421 2028 630, Fax: +49 (0)421 2028 690, Email: wmohr@mpi-bremen.de

Supplementary Table S1: Molar C:P and N:P ratios of cellular biomass. Median C:P and N:P ratios (± standard deviation) were measured using EDS at the start (0 h) and end (24 h) of the incubation on 08 August 2015. Numbers in parentheses indicate the number of measured cells. All changes within 24 hours were significant for all three cyanobacteria (Kruskal-Wallis test (non-parametric), $p < 0.05$).

Organism	Molar C:P (0 h)	Molar C:P (24 h)	Molar N:P (0 h)	Molar N:P (24 h)
Aphanizomenon	124 ± 144 (98)	159 ± 879 (52)	6.7 ± 9.9 (98)	20.4 ± 23.7 (52)
Dolichospermum	134 ± 33 (96)	180 ± 117 (97)	11.3 ± 3.9 (96)	22.4 ± 16.3 (97)
Nodularia	123 ± 27 (72)	179 ± 80 (63)	5.8 ± 2.3 (72)	15.9 ± 8.1 (63)

Supplementary Figures

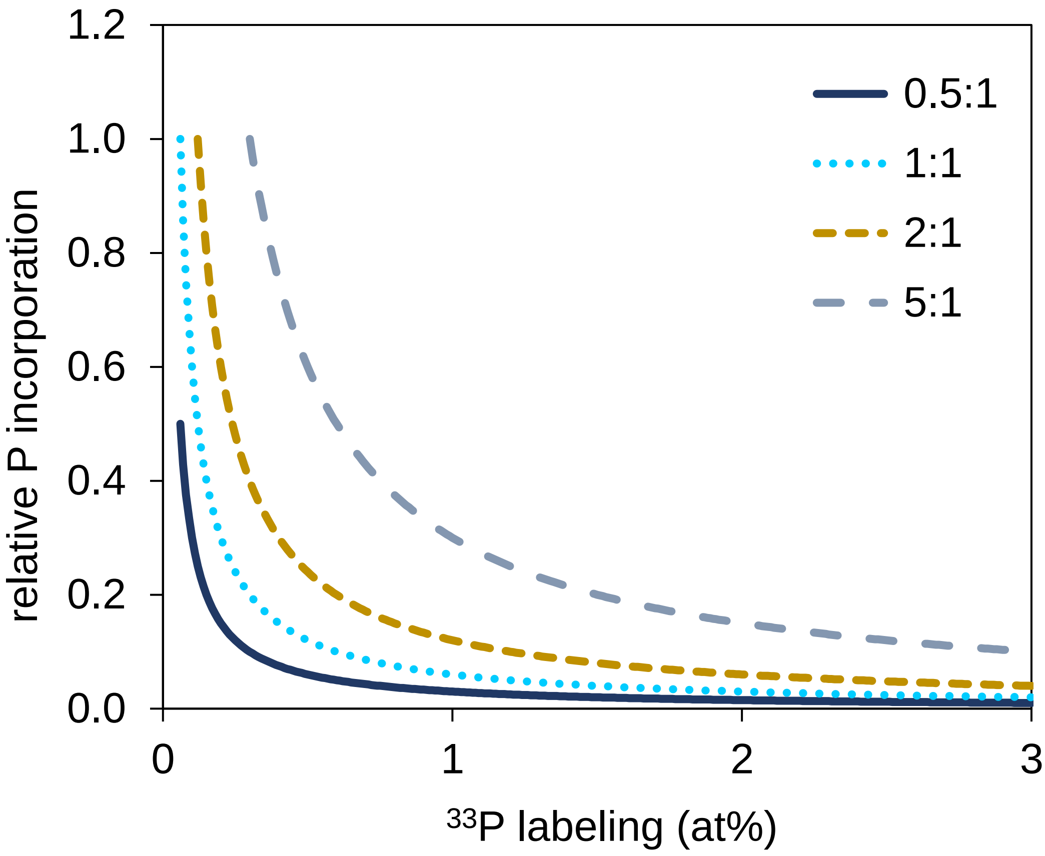

Supplementary Figure S1: Detection limit of relative DIP incorporation. The detection limit of the relative P incorporation varies with the ^{33}P labeling percentage (i.e. the excess ^{33}S at% above the natural abundance of 0.75 at%) and the cellular S:P ratio since the incorporated ^{33}P mass needs to exceed the natural abundance of ^{33}S to the degree that the ^{33}S/^{32}S ratio is above the detection limit (as stated in the methods section). In our experiments, cellular S:P ratios were around 0.5:1 and detection limits were 0.107 and 0.012 in June and August, respectively. For example, the detection limit of 0.012 indicates that at least 1.2 % of the cellular P needs to be newly incorporated P so that the uptake rate is detectable using the newly-developed method.

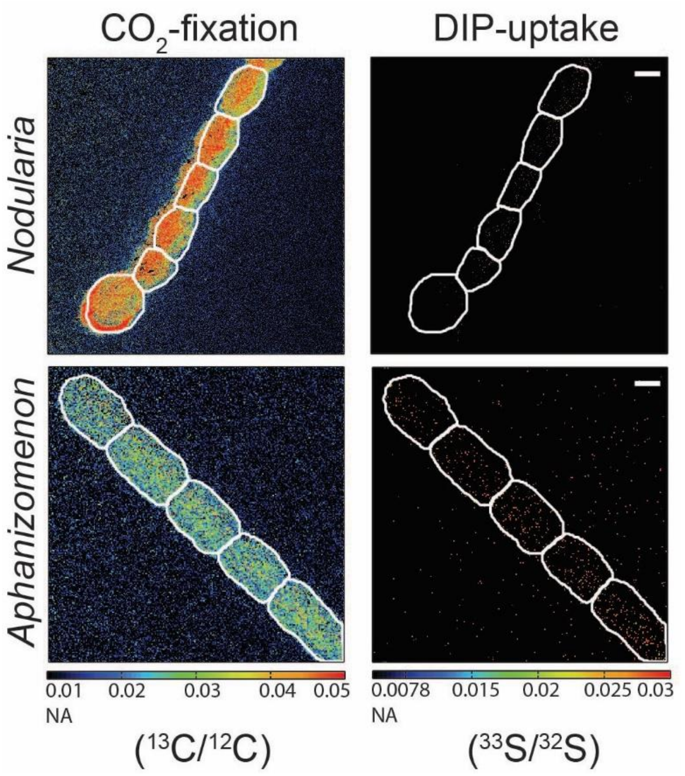

Supplementary Figure S2: Single-cell imaging of cyanobacterial filaments unenriched in ^{33}S. Example images show that not all cells, although active in $^{13}CO_2$ fixation, had detectable DIP uptake as indicated by the $^{33}S/^{32}S$ ratios measured via nanoSIMS in filaments of a *Nodularia* culture and filaments of *Aphanizomenon* collected in June from the Baltic Sea, both under sufficiently high DIP concentrations (same incubations as cells and measurements in Figures 1 and 2). White outlines indicate the cyanobacterial cells. NA = natural abundance. Scale bars are 3 µm for all images.

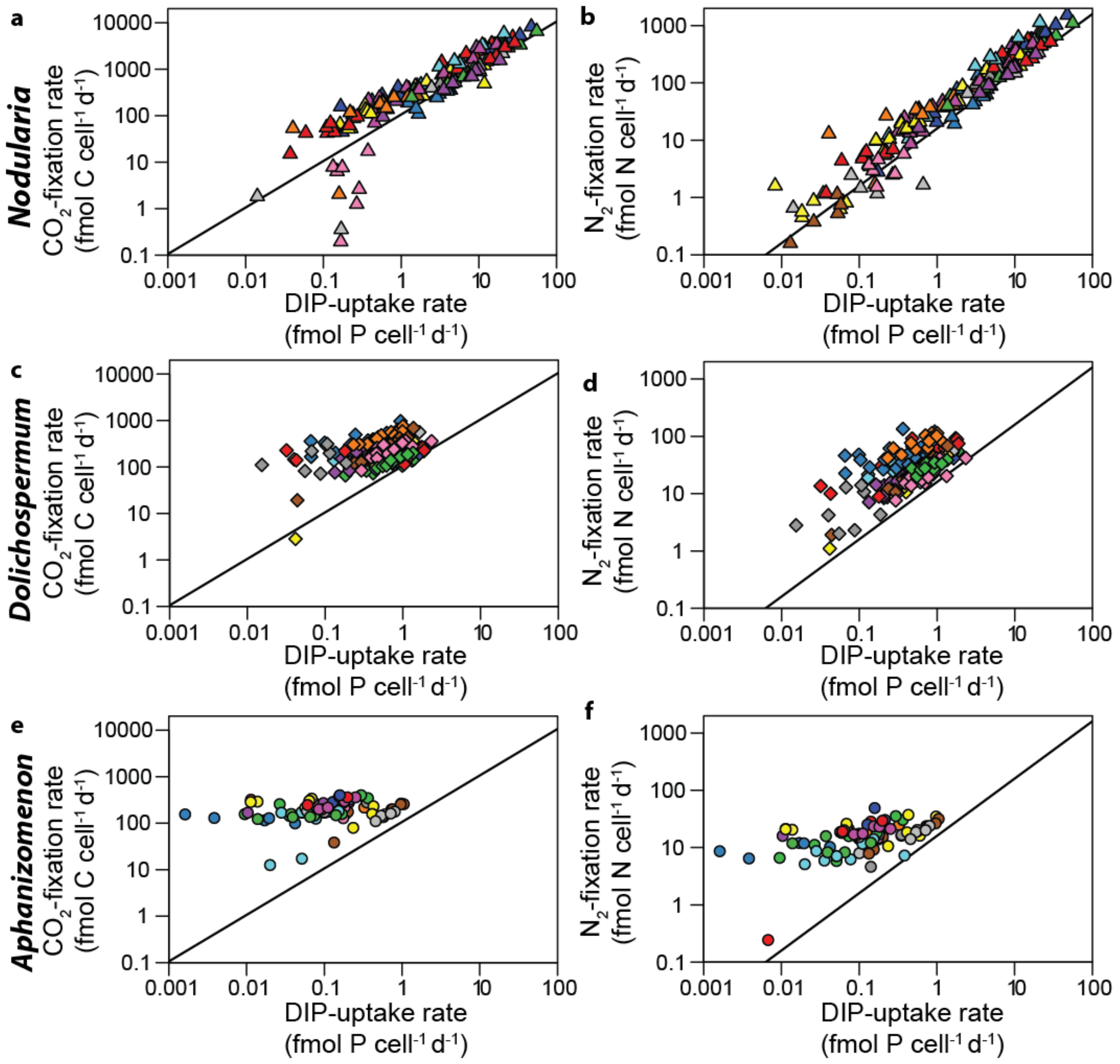

Supplementary Figure S3: Single-cell activity measurements within filaments. CO_2 fixation vs DIP uptake rates (a, c, e) and N_2 fixation vs. DIP uptake rates (b, d, f) for *Nodularia* (a, b), *Dolichospermum* (c, d) and *Aphanizomenon* (e, f) in August 2015. Cells from the same filament share the same color within each panel, showing the phenotypic heterogeneity within a filament that often spans one or more orders of magnitude.

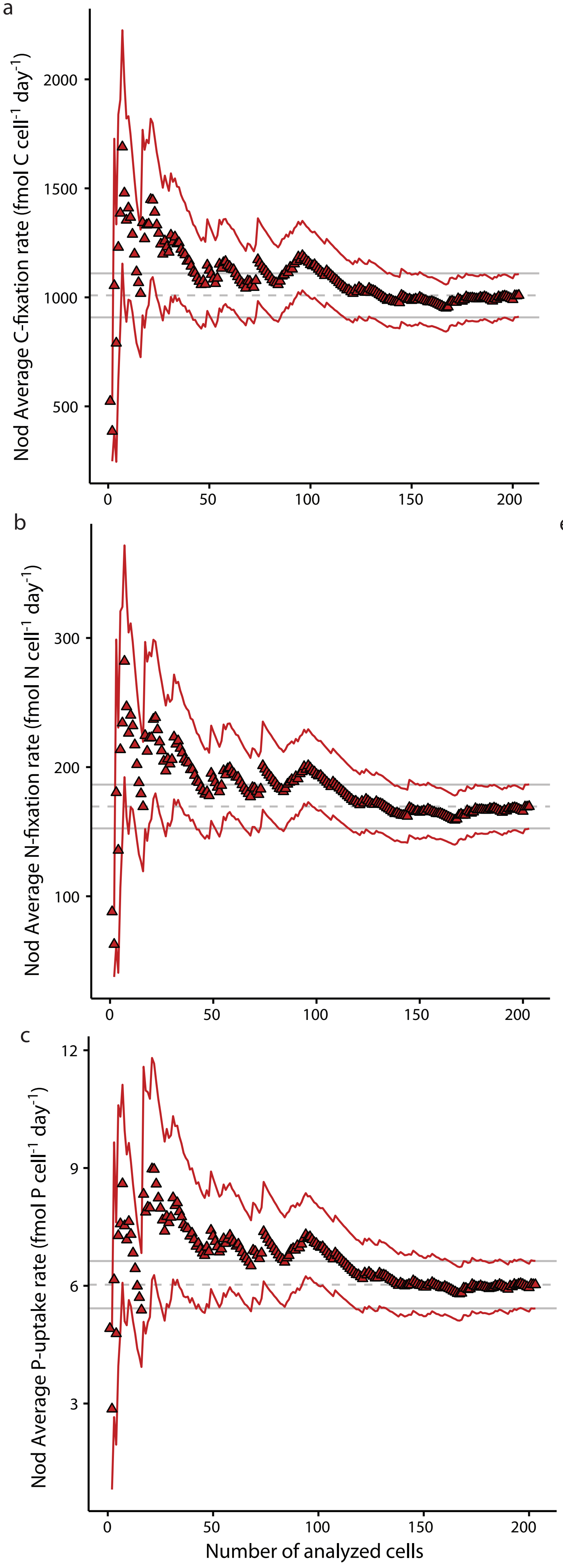

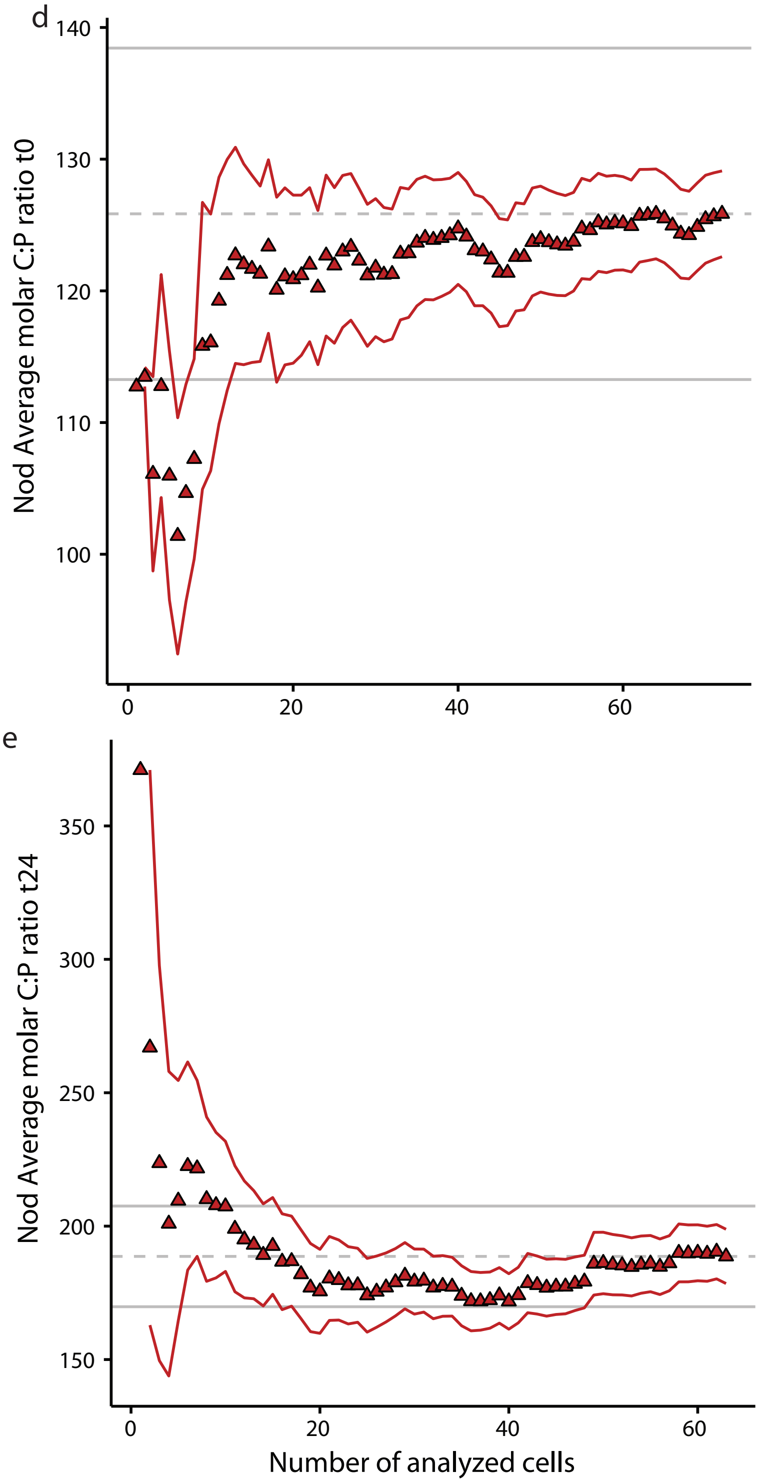

Supplementary Figure S4: Statistical analysis of single-cell rates of *Nodularia*. Symbols and solid lines represent the means and standard errors of CO_2 fixation (a), N_2 fixation (b), DIP uptake (c) rates and the molar C:P ratios at t0 (d) and t24 (e) calculated across randomly subsampled cells. The grey dotted line represents the mean across all cells, the solid grey lines ± 10% of the mean.

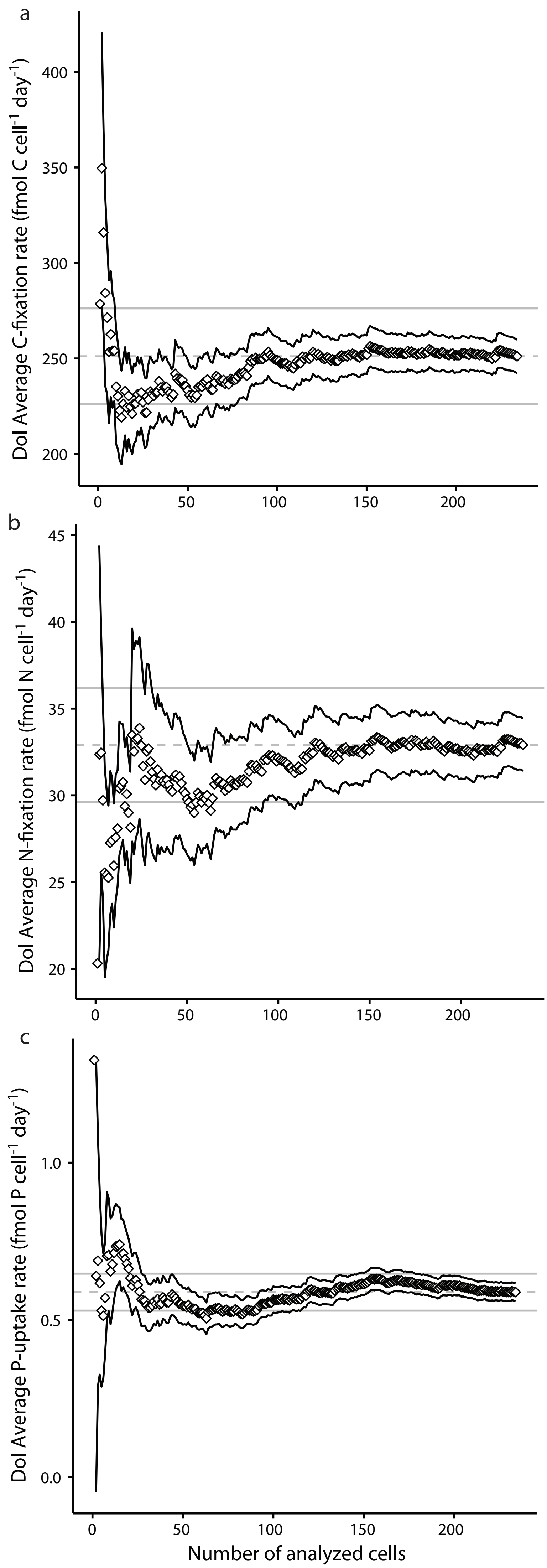

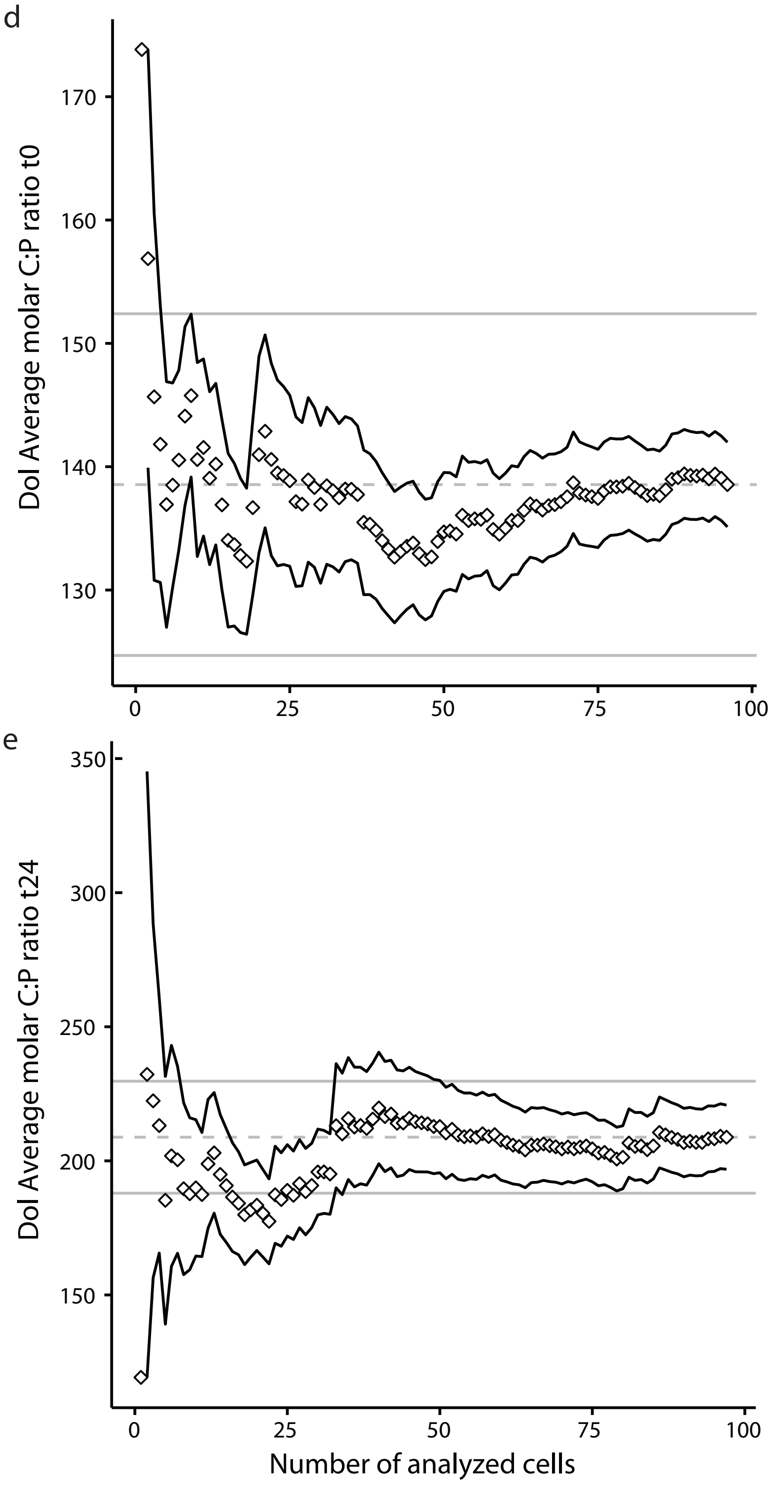

Supplementary Figure S5: Statistical analysis of single-cell rates of *Dolichospermum*. Symbols and solid lines represent the means and standard errors of CO_2 fixation (a), N_2 fixation (b), DIP uptake (c) rates and the molar C:P ratios at t0 (d) and t24 (e) calculated across randomly subsampled cells. The grey dotted line represents the mean across all cells, the solid grey lines ± 10% of the mean.

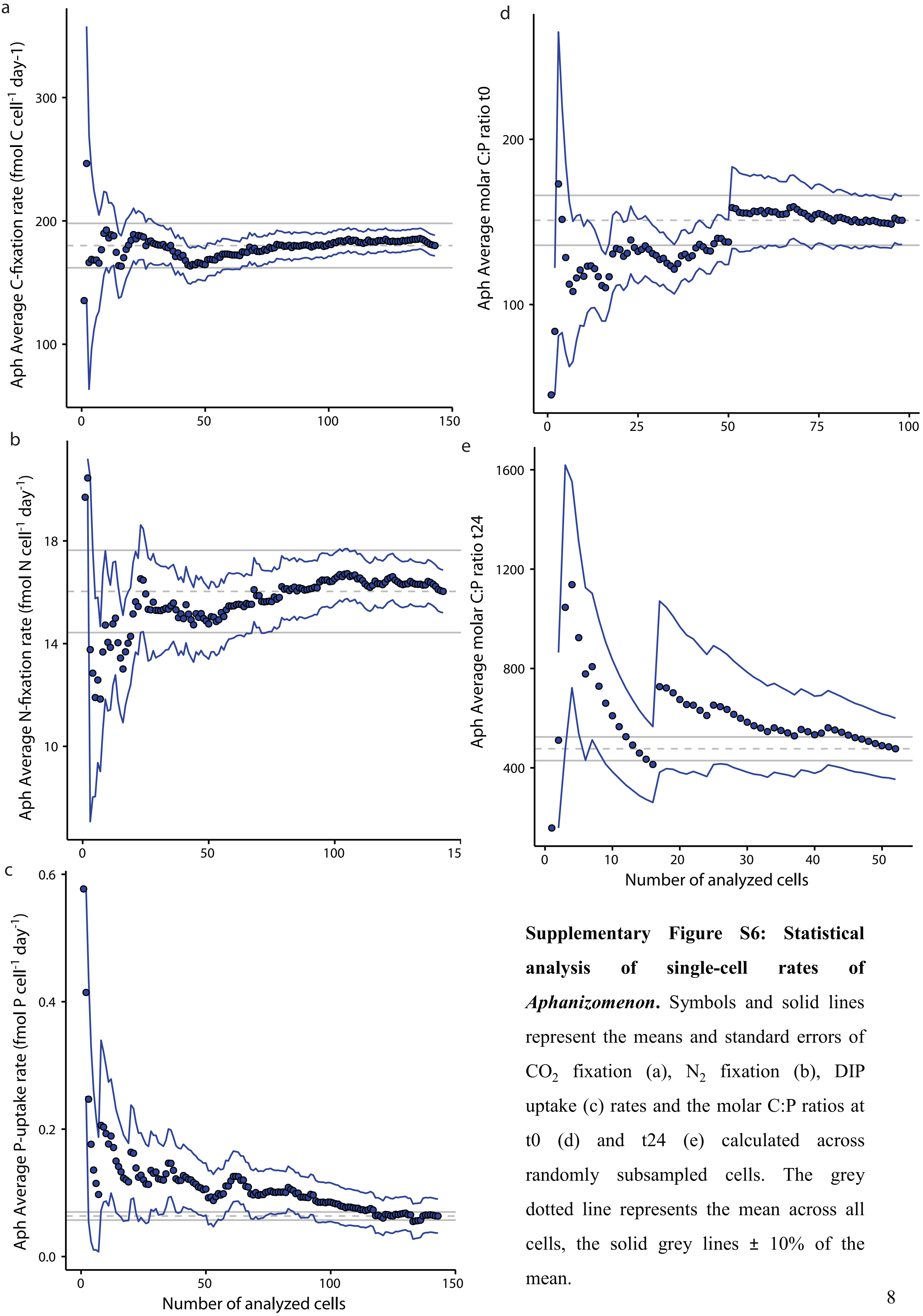

Supplementary Figure S6: Statistical analysis of single-cell rates of *Aphanizomenon*. Symbols and solid lines represent the means and standard errors of CO_2 fixation (a), N_2 fixation (b), DIP uptake (c) rates and the molar C:P ratios at t0 (d) and t24 (e) calculated across randomly subsampled cells. The grey dotted line represents the mean across all cells, the solid grey lines ± 10% of the mean.

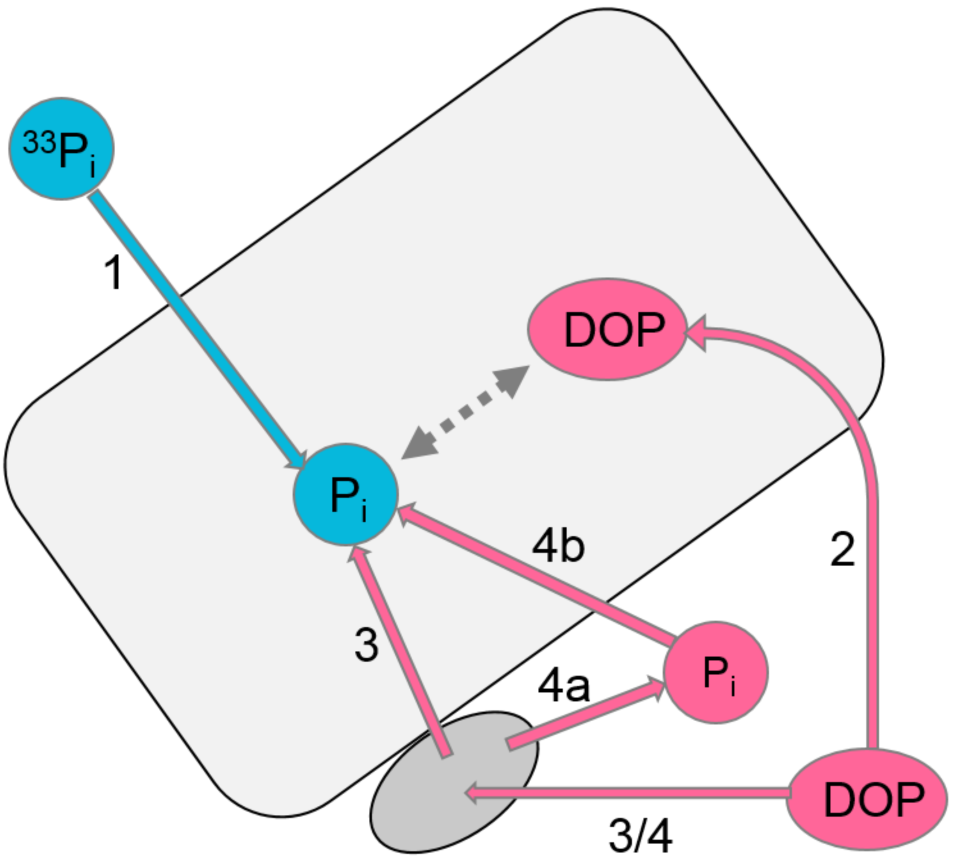

Supplementary Figure S7: Conceptual diagram of DIP and DOP uptake and recycling by a cyanobacterial cell and its microbiome. Direct uptake of DIP from the initial ^{33}P-labeled DIP pool (1) by the cyanobacterial cell is measured by our ^{33}P-DIP method (blue arrow). Direct DOP uptake (including periplasmic or other membrane-bound phosphatases or DOP transport into the cytoplasm; 2), cleavage of P_i from DOP by the microbiome and the direct (3) or indirect (4) transfer of that P_i to the cyanobacterium are defined as DOP usage. Cyanobacterial cell = light grey, epibiont = dark grey, ^{33}P-DIP (P_i) uptake = blue arrow, DOP usage = pink arrows.
